# Vascular-targeted photodynamic therapy with BF2-chelated Tetraaryl-Azadipyrromethene agents: a multi-modality molecular imaging approach to therapeutic assessment

**DOI:** 10.1038/sj.bjc.6605247

**Published:** 2009-10-13

**Authors:** A T Byrne, A E O'Connor, M Hall, J Murtagh, K O'Neill, K M Curran, K Mongrain, J A Rousseau, R Lecomte, S McGee, J J Callanan, D F O'Shea, W M Gallagher

**Affiliations:** 1 UCD School of Biomolecular and Biomedical Science, UCD Conway Institute, University College Dublin, Belfield, Dublin 4, Ireland; 2 Department of Physiology and Medical Physics, Royal College of Surgeons in Ireland, Reservoir House Sandyford Industrial Estate, Ballymoss Road, Dublin 18, Ireland; 3 Centre for Synthesis and Chemical Biology, University College Dublin, Belfield, Dublin 4, Ireland; 4 UCD School of Medicine and Medical Science, University College Dublin, Belfield, Dublin 4, Ireland; 5 Sherbrooke Molecular Imaging Centre, Etienne Le-Bel Clinical Research Centre, Centre Hospitalier Universitaire de Sherbrooke and Université de Sherbrooke, Sherbrooke, Quebec, Canada; 6 UCD School of Agriculture, Food Science and Veterinary Medicine, Veterinary Science Centre, University College Dublin, Belfield, Dublin 4, Ireland

**Keywords:** BF_2_-chelated tetraaryl-azadipyrromethene, photodynamic therapy, vascular targeting, biodistribution, molecular imaging, drug efficacy

## Abstract

**Background:**

Photodynamic therapy (PDT) is a treatment modality for a range of diseases including cancer. The BF_2_-chelated tetraaryl-azadipyrromethenes (ADPMs) are an emerging class of non-porphyrin PDT agent, which have previously shown excellent photochemical and photophysical properties for therapeutic application. Herein, *in vivo* efficacy and mechanism of action studies have been completed for the lead agent, ADMP06.

**Methods:**

A multi-modality imaging approach was employed to assess efficacy of treatment, as well as probe the mechanism of action of ADPM06-mediated PDT.

**Results:**

Tumour ablation in 71% of animals bearing mammary tumours was achieved after delivery of 2 mg kg^−1^ of ADPM06 followed immediately by light irradiation with 150 J cm^−2^. The inherent fluorescence of ADPM06 was utilised to monitor organ biodistribution patterns, with fluorescence reaching baseline levels in all organs within 24 h. Mechanism of action studies were carried out using dynamic positron emission tomography and magnetic resonance imaging techniques, which, when taken together, indicated a decrease in tumour vascular perfusion and concomitant reduction in tumour metabolism over time after treatment.

**Conclusion:**

The encouraging treatment responses *in vivo* and vascular-targeting mechanism of action continue to indicate therapeutic benefit for this new class of photosensitiser.

Photodynamic therapy (PDT) is an emerging treatment modality whereby a photosensitiser is administered systemically or locally and subsequently activated by illumination with visible light, leading to the generation of cytotoxic reactive oxygen species (Palumbo, 2007). The interaction of photosensitiser and light results in photochemical production of activated oxygen species, from endogenous oxygen, which attack key structural entities within the targeted cells. Photodynamic therapy is currently used for the treatment of various types of cancer, including the lung, skin, gastrointestinal tract, head and neck, and urological cancers, as well as non-cancer diseases such as age-related macular degeneration (AMD), atherosclerosis, and viral or bacterial infections (Kossodo and LaMuraglia, 2001; Hamblin and Hasan, 2004; Wormald *et al*, 2007). The efficacy of PDT in the treatment of cancer depends on photosensitiser concentration and localisation, coupled with light dose (fluence), and oxygen availability. The singlet oxygen generated by the photochemical reaction of photosensitiser and light can directly kill tumour cells through the induction of apoptosis and/or necrosis, or may damage the tumour vasculature and healthy vessels, resulting in hypoxia and oxygen starvation, thus causing indirect tumour kill (Triesscheijn *et al*, 2006). In addition, PDT can initiate an immune response against the remaining tumour cells. The outcome of PDT is dependent on all these mechanisms and the relative contribution of each depends on the treatment regimen employed (Triesscheijn *et al*, 2006).

To elicit a vascular-targeting effect, therapeutic effectors or cytotoxic agents need to be selectively delivered to the tumour vasculature (Chen *et al*, 2006). Passive targeting of the tumour vessels while an i.v. injected agent is still within the vessel is perhaps the most feasibly effective approach. During recent years, vascular-targeted PDT has received much interest and select vascular-targeting photosensitisers are already in clinical development for PDT of AMD and certain cancers (Trachtenberg *et al*, 2007, 2008; Schmidt-Erfurth and Sacu, 2008). The relative contributions of vascular-mediated *vs* direct tumour toxicity depend on the sensitiser and on the interval between sensitiser administration and illumination. For short intervals (i.e., irradiation directly after drug administration), when there is a high drug concentration in circulating plasma, the vascular damage is maximised. For longer intervals, when the drug has been distributed to tumour tissue, direct cell toxicity becomes more important (Chen *et al*, 2006).

We have previously reported the discovery of a novel class of non-porphyrin-based modifiable PDT agents, termed the BF_2_-chelated tetraaryl-azadipyrromethenes (ADPMs), and have shown *in vitro* that their photophysical and biological characteristics have significant potential for the development as new anti-cancer therapeutics (Gorman *et al*, 2004; Gallagher *et al*, 2005). We have shown that this new class of PDT agent possesses excellent photophysical properties and as having EC_50_ values in the low nano-molar range for lead compound ADPM06, with no discernable activity bias for a specific tumour cell type. Moreover, these novel photosensitisers show low to non-determinable dark toxicity. Critically, the synthesis of these compounds is straightforward and results in defined single agents. This characteristic places the ADPM genus in the category of being a family of pure and modifiable molecular photosensitisers. Thus, the ADPMs are discrete structures that are amenable to modification around the periphery of the chromophore, thus allowing for optimisation of all aspects of their photophysical and therapeutic properties.

In this study, we have used a short drug–light interval protocol with lead compound ADPM06 to show tolerance and efficacy in several animal models of cancer. We have also exploited the inherent fluorescent properties of a candidate PDT agent to directly assess *in vivo* drug biodistribution profiles using optical imaging. Moreover, using positron emission tomography (PET) and magnetic resonance imaging (MRI), we have also shown that our current ADPM06 treatment regimen elicits a vascular-targeting effect.

## Materials and methods

### Photosensitiser

The synthesis and formulation of the ADPM family of compounds in a mixture of Cremophor EL (CrEL)/1,2-propanediol (10 : 3 v/v) has been described previously (Gorman *et al*, 2004). The quantity of CrEL/1,2-propanediol in an assayed photosensitiser solution was always <0.03%.

### Cells

MDA-MB-231 tumour cells were obtained from the American Type Culture Collection (ATCC) and cultured in minimum essential medium supplemented with 10% (v/v) foetal calf serum, 1% (v/v) non-essential amino acids, 50 U ml^−1^ penicillin, 50 *μ*g ml^−1^ streptomycin, 1% (v/v) sodium pyruvate, and 1% (v/v) L-glutamine. All cells were maintained in 5% CO_2_ (v/v) and 21% O_2_ (v/v) at 37°C. MDA-MB-231 luciferase-expressing cell line (MDA-MB-231-luc) was obtained from Caliper LS (Alameda, CA, USA) and maintained as above. Human umbilical vein endothelial cells (HUVECs) were obtained from Clonetics (Basel, Switzerland) and maintained in endothelial cell growth medium (EGM; Clonetics), containing 12 *μ*g ml^−1^ bovine brain extract, 10 ng ml^−1^ EGF, 1 *μ*g ml^−1^ hydrocortisone, 50 *μ*g ml^−1^ gentamicin, 50 ng ml^−1^ amphotericin B, and 2% foetal bovine serum (Clonetics).

### Stable expression of GFP reporter through lentiviral transduction

A stable MDA-MB-231 breast cancer cell line capable of constituitive, enhanced GFP expression was generated through lentiviral transduction. For this, viral particles were produced by transient calcium phosphate transfection of HEK 293t cells with the GFP-encoding lentivector, pLVTHm, the envelope vector, pMD2G, and the packaging vector, psPAX2 (all vectors were obtained from D Trono, University of Geneva, Switzerland). Transfected cells were incubated for 48 h, after which medium containing the lentiviral particles was harvested and passed through a 0.45-*μ*m filter. Viral supernatant was placed directly on subconfluent MDA-MB-231 cells, which were then incubated overnight before the cells were washed gently with sterile PBS and the media was refreshed.

### *In vitro* angiogenesis assay

Tube formation assays were performed using an *in vitro* Angiogenesis Assay Kit (Chemicon International, Inc., Temecula, CA, USA). Briefly, solid gels were prepared according to the manufacturer's instructions on a 96-well tissue culture plate. HUVECs seeded into 25-cm^2^ flasks and grown to confluency of 70–80%, were irradiated 3 h after ADPM06 administration (150 nM). Cells were then trypsinised and seeded at a density of 5 × 10^4^ cells per well in EGM onto the surface of the solid endothelial cell gel matrix. The cells were incubated for 7 h at 37°C in a CO_2_ incubator. Tube formation was observed under an inverted light microscope at × 20 magnification. Microscopic fields were photographed with a digital camera. The number of branch-points per field-of-view was counted, and the length of tubules was quantified using ImageJ 1.41 (National Institutes of Health, Bethesda, MD, USA). Mann–Whitney *U*-test was used to compare control and treated samples.

### Animal models

All animal experiments were licensed by the Department of Health and Children, Ireland and specific protocols reviewed by the Animal Research Sub Committee (ARSC) at University College Dublin. Imaging protocols were conducted in accordance with the recommendations of the Canadian Council on Animal Care and of the in-house Ethics Committee for Animal Experiments at Université de Sherbrooke. All studies were in full compliance with EU guidelines. Female C57/Bl or Balb C nu/nu mice were received to the SPF-grade Conway Institute Biotechnical Services (CIBS) Xenograft Facility at 4–6 weeks old (Harlan, UK). Initially, normal C57/Bl mice were parenterally treated (i.v.) with ADPM06 (1–10 mg kg^−1^) to establish a basic toxicity profile for the novel photosensitiser. There was no evidence of either acute or chronic (6-month follow-up) toxicity. No significant histopathological changes were observed on veterinary pathologist's examination of the liver, kidney, heart, or spleen. Specifically, no necrotic, degenerative, inflammatory, or hyperplastic processes were observed (data not shown). For light+drug toxicology studies (as well as drug efficacy studies), C57/Bl mice were shaved on the right hind limb and depilated with Nair (Carter-Wallace Inc., New York, NY, USA). Mice were anesthetised with an i.p. injection of ketamine/xylazine cocktail (90 mg kg^−1^ ketamine and 10 mg kg^−1^ xylazine). One million LLC/1 lung carcinoma cells were injected subcutaneously in one mid-thigh area suspended in 100 *μ*l PBS. Tumours grew predictably in all the mice and reached a size of 5- to 6-mm diameter 5–7 days after injection, at which time they were used for PDT. Female Balb C nu/nu mice were similarly anesthetised and five million MDA-MB-231-GFP or MDA-MB-231-luc cells were injected subcutaneously in 100 *μ*l PBS/Matrigel (50 : 50) into the right forelimb. Both cell lines similarly reached 5–6 mm in diameter within 7–10 days and were subjected to PDT as described below. For dynamic PET and MRI studies, MAT III B tumour cells (obtained from ATCC) were inoculated intradermally (5 × 10^6^ cells in PBS) into both axillary areas on rats 6–8 days before the experiment. Rats were fasted 12 h before PDT treatment.

### Drug efficacy studies: PDT protocol

ADPM06 in PBS/Cremophor solvent was injected at a dose range of 1–10 mg kg^−1^ in 0.3 ml solution through the lateral tail vein. The PDT was performed immediately after injection using a Lumacare LC-122M fibre-optic light delivery system (Lumacare, Newport Beach, CA, USA) emitting light at 690 nm (±25 nm). The illuminating spot had a diameter of 11 mm and was positioned so that the entire tumour and a surrounding 2–3 mm area of normal tissue were exposed to light with no evident temperature increase at the site of irradiation. Mice were anesthetised as described above and the tumour-bearing limb positioned under the spot. Total fluences between 50 and 200 J cm^−2^ were delivered at a fluence rate of 717 mW cm^−2^. Some mice had LLC/1, MDA-MB-231-luc, or MDA-MB-231-GFP tumours illuminated without having received an earlier injection of ADPM06. In addition, another group of animals were treated with ADPM06 i.v. in the absence of light. At the completion of illumination, mice were allowed to recover in an animal warmer until they resumed normal activity. A positive tumour response was assigned to tumours that appeared macroscopically as flat blackened eschars and necrotic tissue within a few days after PDT. Animals were considered cured after complete tumour regression; this was defined as the absence of a palpable tumour.

### Dynamic PET and MRI studies: PDT protocol

Rats bearing 13762 MAT B III tumours were anesthetised and two cannulas were inserted in the caudal veins, one for radiotracer infusion or contrast agent injection (MRI) and the other for photosensitiser administration. The animals were then placed on the scanner bed equipped with a heat pad and instrumented for life sign monitoring. For the dynamic PET studies, 0.8 mg kg^−1^ of the photosensitiser was administrated to rats lying in the scanner through a second cannula 30 min after the commencement of ^18^F-FDG infusion. Illumination was provided immediately after the photosensitiser injection (less than 5 min between photosensitiser injection and tumour illumination). One tumour was illuminated with a 670-nm light beam delivered through a fibre optic by a diode laser (model BWF2-692-0.7-100-0.22-SMA; B&W Tek, Inc., Newark, NJ, USA) and the other tumour was masked and served as control. The light beam was spread uniformly over the whole tumour area and maintained for 215 s at a fluence rate of 700 mW cm^−2^ for a total fluence of 150 J cm^−2^, as before. For PET and MRI imaging studies at specific time points before or after PDT, the photosensitiser administration and tumour illumination were performed off the scanner after the same treatment protocol as above.

### Optical imaging

Optical imaging was performed with an IVIS Spectrum small-animal *in vivo* imaging system (Caliper LS), having both bioluminescence and fluorescence capabilities. Images and measurements of fluorescent signals were acquired and analysed using Living Image Software v3.0 (Caliper LS). Excitation and emission wavelengths of 475 and 540 nm, respectively, were used for acquiring *in vivo* GFP fluorescent images, whereas excitation and emission wavelengths of 640 and 720 nm, respectively, were used for acquiring *in vivo* ADPM06 fluorescent images. All images were attained using a 1-s exposure time and an f/stop of 1, with a sampling of multiple angles with animal remaining sedated. For biodistribution studies, mice were injected with 2 mg kg^−1^ ADPM06 through the tail vein, anesthetised before being imaged at ADPM06 excitation and emission wavelengths at various time points. Mice were then euthanized, the tumour, liver, lungs, heart, spleen and kidneys were extracted, and *ex vivo* fluorescence images were acquired. MDA-MB-231-luc tumours were imaged following i.p. administration of luciferin substrate (150 mg kg^−1^).

### PET imaging

PET imaging was performed with the Sherbrooke avalanche photodiode PET scanner (University of Sherbrooke, Quebec, Canada) (Lecomte *et al*, 1996), achieving 2.1 mm resolution in-plane (14 *μ*l volumetric resolution). The scanner is made of four detector rings with a total of 512 pixel detectors defining an axial field-of-view of 2.5 cm with a diameter up to 10 cm. The massively parallel processing of pixel detectors makes it possible to image over a large range of radioactivity with negligible dead time losses. Therefore, dynamic PET imaging of tumour ^18^F-FDG uptake during PDT treatment could be performed with radiotracer infusion for up to 4 h, starting at the same time as the dynamic image acquisition. Rats were typically infused at 0.008 ml min^−1^ with an initial ^18^F-FDG radiotracer concentration of ∼1.5 mCi ml^−1^. Tumours were positioned at the centre of the scanner axial field-of-view in such a way to allow illumination of one tumour with the laser light during the scan, whereas the other one (used as control) was masked. Rats were anesthetised using a mix of 2% isofluorane in oxygen with the flow set at no more than 2 ml min^−1^ during these extended scans and the animal vital signs were monitored and recorded throughout the entire scan time to ensure a stable physiological status at all times and for later reference. Scanning was stopped if vital signs were not at physiological status. The list mode PET data were sorted out into 1-min frames and PET images were reconstructed using 10 iterations of a maximum likelihood expectation maximisation (MLEM) algorithm that models detector response. Image series consisted of up to 240 1-min frames. These PET measurements were performed with PDT treatment (*n*=5), with ADPM06 alone (*n*=1) and light alone (*n*=1). Regions of interest (ROIs) were traced over the treated and the control tumours using the last frame of the dynamic image series and applied to all frames. Time-activity curves corrected for radioactive decay were generated from the maximum counts averaged over 4 pixels in the ROIs. Taking the control tumour as reference, the relative metabolic activity of the treated and control tumours was estimated as the ratio of the slope of these two curves. Mean group values were tested for statistical significance using a one-way analysis of variance. The PET images of the tumours after a bolus injection of 1–1.3 mCi ^18^F-FDG (in <1 min) were also obtained at specific time points before PDT and at 30 min, 24 and 48 h after PDT treatment to monitor tumour metabolism over a more extended period of time by simply comparing ^18^F-FDG uptake values at equilibrium in tumour ROIs. A static 1-min image frame at 30 min after the bolus injection was extracted from the list mode data and reconstructed using 10 MLEM iterations. The relative metabolic activity of the treated and control tumours were also estimated by ROI analysis as described above.

### Magnetic resonance imaging

The MRI was performed with a Varian 7T small animal scanner (Varian Inc., Palo Alto, CA, USA) using a 63-mm volume coil. Rats were anesthetised using a mix of 2% isofluorane in oxygen with the flow set at 2 ml min^−1^ and were restrained to prevent movement during the experiment. Dynamic acquisition was started 3, 24, and 48 h after PDT (*T*_1_-weighted images, repetition time=210 ms, echo time=2.49 ms, field-of-view 4 × 5 cm^2^, matrix=128 × 128, flip angle=30°, four averages, 20 slices of 1.5 mm). Sets of images were acquired continuously. A bolus of 0.6 ml gadolinium diethylenetriamine pentaacetic acid (Gd-DTPA) (Magnevist, Berlex Canada Inc., Pointe Claire, QC, Canada) was injected i.v. 5 min after the start of the scan (with an infusion rate of 600 *μ*l min^−1^) through the tail vein after the second set of images.

## Results

### *In vitro* effects of ADMP06-mediated PDT on HUVECs

**Figure 1:**
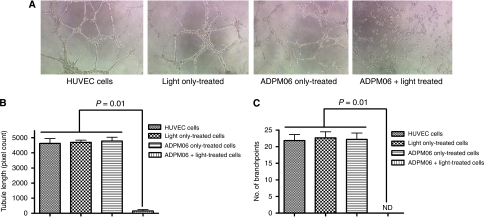
*In vitro* endothelial cell tubule formation assay. (**A**) The HUVECs, post-treatment with ADMP06-mediated PDT, were seeded in Matrigel-coated plates and photographed 7 h post-seeding. Images shown for PDT treatment (150 nM ADPM06+16 J cm^−2^ light) compared with cells alone, light alone, and drug alone controls. Experiment was carried out in triplicate, with representative images shown. Image analysis determined the total tubule length (**B**), and the number of branch-points per junction (**C**) in each sample. The Mann–Whitney *U*-test was used to compare control and treated samples. (ND, non-determinable).

The capacity of HUVECs to form new tubules in Matrigel (BD Biosciences, Bedford, MA, USA) was directly inhibited after PDT with ADPM06 (Figure 1), indicative of an anti-angiogenic effect. No effects on tubule length (Figure 1B) or branch-point formation (Figure 1C) were seen on cells either irradiated with light alone, or treated with drug alone.

### *In vivo* response to ADPM06-mediated PDT delivered with short (immediate) drug–light interval

**Figure 2:**
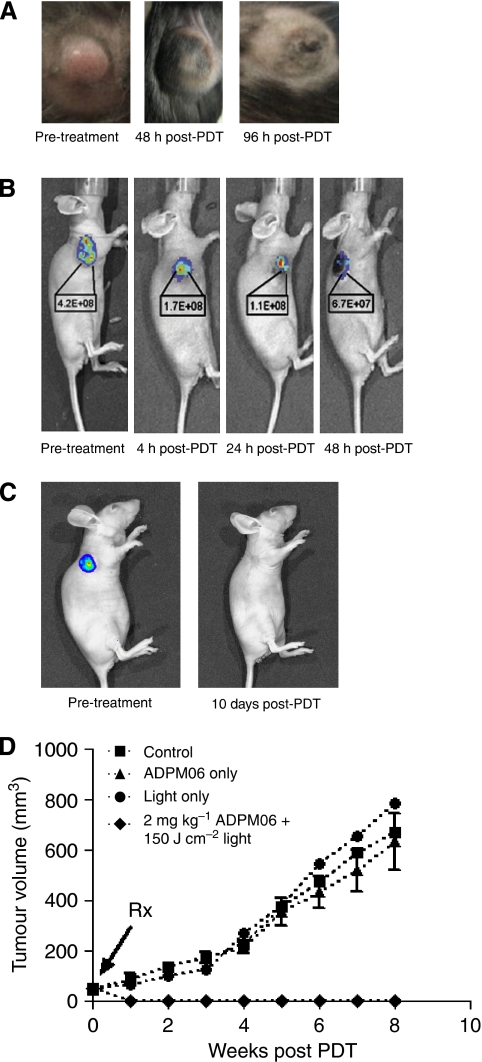
*In vivo* response to ADPM-mediated PDT delivered with short drug–light interval. (**A**) Photographs of LLC/1 syngeneic murine tumour model before, 48 and 96 h after treatment (2 mg kg^−1^ ADPM06+150 J cm^−2^ light). (**B**) and (**C**) MDA-MB-231-GFP xenograft tumour ablation after treatment (2 mg kg^−1^ ADPM06+150 J cm^−2^ light) as shown by reduction in measurable tumour fluorescence. Representative images of a single animal from four repeat studies. Decreasing fluorescence intensity values are shown (photons per second per cm^2^ per steradian). (**D**) ADPM06 efficacy study in MDA-MB-231-GFP xenograft model. Treatment groups were as follows: PDT group (2 mg kg^−1^ ADPM06 IV+150 J cm^−2^ light (*n*=7)), light-alone group (150 J cm^−2^ (*n*=8)), drug-alone group (2 mg kg^−1^ ADPM06 i.v. (*n*=14)), and vehicle control (*n*=9).

LLC/1 tumour-bearing mice were used to determine drug–light combination tolerance. Initial studies comparing toxicity profiles of ADPM06 in combination with light (70–300 J cm^−2^) provided early evidence of tumour response (Figure 2A). Mass tumour necrosis was evident within 72–96 h and an optimal light fluence of 150 J cm^−2^ was determined. After PDT, oedema and inflammation became apparent by 1–2 days, with the subsequent development of necrosis and eschar by days 3–5, but confined to the tumour region. Oedema that healed within a few days leaving no tissue damage was observed in the illuminated normal tissue around the tumour. Some erythema (that cleared within the same time) in the immediately adjacent, non-illuminated, normal tissue was invariably observed. In contrast, tumour healing took approximately 20–30 days. The skin covering the tumour was initially damaged, but underwent remodelling during the healing process.

For xenograft studies, MDA-MB-231-GFP cells inoculated subcutaneously into nude mice developed tumours. Within 7–10 days, when the tumours reached a diameter of about 7–9 mm, PDT treatment was performed, using different light–drug concentrations. In all cases, tumours were irradiated immediately after i.v. drug delivery. The illumination of the tumour always included 2–3 mm of surrounding normal tissue. Tumour ablation over time, as shown by reduction in GFP fluorescence, is shown in Figure 2B and C. All animals at drug–light doses of 2 mg kg^−1^ ADPM06+100–200 J/cm^2^ light responded to therapy. Cure was defined as the disappearance of tumour with no recurrence at 6 months. All animals tolerated 2 mg kg^−1^ ADPM06+150 J/cm^2^ light well, resulting in a high tumour cure rate of 71%. In total, 45% of animals treated with 2 mg kg^−1^ ADPM06+100 J cm^−2^ light were cured, 33% with 2 mg kg^−1^ ADPM06+50 J cm^−2^ light and 27% with 1 mg kg^−1^ ADPM06+100 J/cm^2^ light. A typical growth curve showing controls *vs* treated animals (2 mg kg^−1^ ADPM06+150 J cm^−2^ light) is shown in Figure 2D.

### Drug biodistribution and clearance as determined by optical imaging

**Figure 3:**
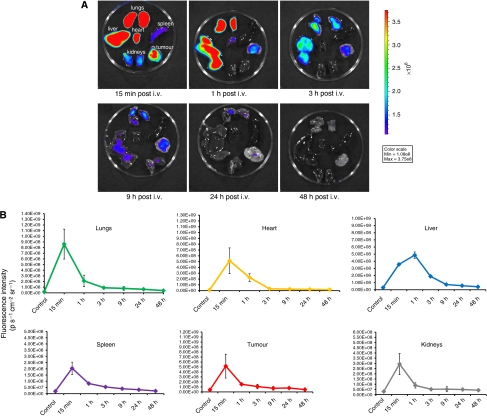

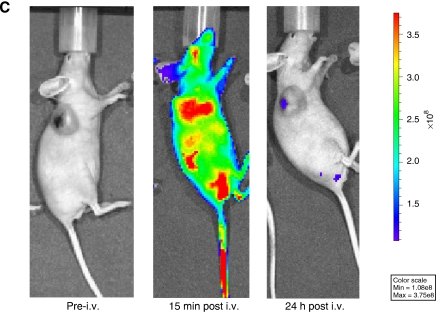
*In vivo* biodistribution of ADPM06 as measured by optical imaging. (**A**) Representative images of excised organs from Balb/C nude mice bearing subcutaneous MDA-MB-231-luc tumours. Mice were killed over a 48-h interval after i.v. injection of ADPM06 (2 mg kg^−1^). Fluorescence intensity peaked in all organs 15 min post injection, with the exception of the liver, which reached maximum fluorescence at 1 h. Fluorescence intensity reached baseline levels by 24 h and seems to be cleared from the animal by 48 h. MDA-MB-231-luc tumour model used in biodistribution studies to prevent GFP autofluorescence into the NIR channel. (**B**) Quantification of fluorescence intensity from the lungs, heart, spleen, tumour, kidneys, and liver over 48 h (photons per second per cm^2^ per steradian). (**C**) *In vivo* fluorescence imaging of Balb C nude mice bearing subcutaneous MDA-MB-231-luc tumours before and after i.v. injection of ADPM06 (2 mg kg^−1^). Animal shown before administration of ADPM06, 15 min post-injection and 24 h post-injection. Fluorescence intensity of ADPM06-treated animals (photons per second per cm^2^ per steradian) was normalised to background fluorescence of animals before ADPM06 administration.

*Ex vivo* imaging and fluorescence quantification from excised organs was performed to establish drug biodistribution patterns (Figure 3A). A peak in fluorescence intensity was observed in the lungs, liver, kidneys, heart, spleen, and tumour within 1 h of drug administration (Figure 3B). Fluorescence within these organs approached baseline levels within 24 h and seemed to be completely cleared from the system by 48 h. Measurable tumour fluorescence decreases at a slower rate compared with the lungs, liver, and kidneys. Comparison of whole body fluorescence associated with ADPM06, 15 min and 24 h post-injection illustrated negligible retention of ADPM06 in the skin (Figure 3C). Critically, drug-associated fluorescence was below system threshold 48 h after parenteral injection.

### PET-based evaluation of ADPM06 effects *in vivo*

**Figure 4:**
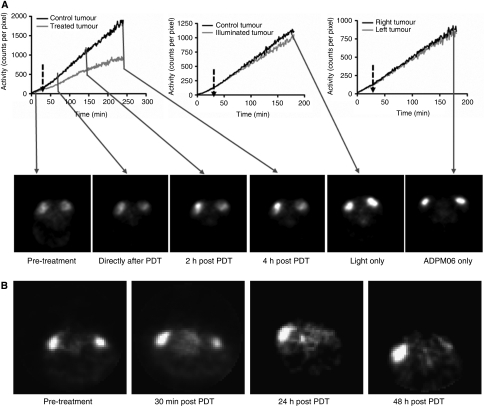
The PET approach to assessing vascular-targeting effect *in vivo.* (**A**) Time-activity curves during ^18^FDG infusion representing dynamic radiotracer uptake in intradermal implanted 13762 MAT B III tumours in PDT (0.8 mg kg^−1^ ADPM06+150 J cm^−2^ light), light alone (150 J cm^−2^), and ADMP06 alone (0.8 mg kg^−1^) treated animals. The delay between ADPM06 injection and illumination commencement was <5 min. ^18^F-FDG PET image slices through tumours taken at selected time points before and after treatment are also shown. (**B**) The PET image slices of tumours after bolus injection of 1–1.3 mCi ^18^FDG and 30 min uptake at selected time points before and after ADPM06-mediated PDT, illustrating the change in tumour metabolic activity. White arrows, control tumour; grey arrows, treated tumours.

Rats bearing 13762 MAT B III tumours receiving ADPM06-mediated PDT were assessed by dynamic PET to investigate the mechanistic basis of this treatment *in vivo* (Figure 4A). Time-activity curves showed a decline in ^18^F-FDG uptake rate in the treated tumour relative to control tumour after light treatment. Photosensitiser and light controls showed no significant modification of the ^18^F-FDG uptake rate relative to the control (untreated) tumour in PDT-treated rats, confirming the absence of any detectable metabolic effects due to ADPM06 or light alone. Therefore, transient changes in the ^18^F-FDG uptake rate, which occurred from 10 to 30 min after the commencement of illumination can clearly be related to the effect of PDT. The subsequent slope change of the ^18^F-FDG uptake curve observed >30 min after PDT is likely the result of the time-confined, short PDT insult causing a partial, but permanent, drop in tumour metabolic activity, specifically 50% (mean±7% *P*<0.05; *n*=5) from the slope value (counts per pixel per minute) of the ^18^F-FDG uptake curves after PDT. Such behaviour does not suggest extensive cellular damage during this observation time window and is consistent with a vascular-targeting response depriving tumour cells of blood supply, when light irradiation is performed immediately after photosensitiser delivery. Bolus tracer injection confirmed reduced tumour metabolism at 30 min post PDT and almost complete suppression of tumour metabolic activity at 24 and 48 h post PDT (Figure 4B and Table 1), which is also consistent with delayed cell death resulting from vascular stasis.

### MRI perfusion studies with ADPM06-mediated PDT

**Figure 5:**
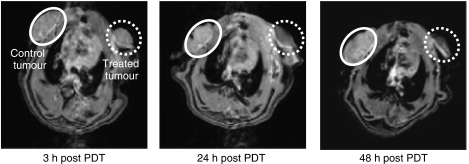
MRI approach to assessing vascular-targeting effect *in vivo.* The MRI of control 13762 MAT B III rat tumour (white circle) and PDT-treated tumour (0.8 mg kg^−1^ ADPM06+150 J cm^−2^ light) (dashed white circle) at ∼3, 24, and 48 h post-treatment with a Gd-DTPA injection, illustrating the change in tumour perfusion.

The MRI was employed to further assess effects of ADPM06-induced PDT on tumour vascular perfusion. Immediately after dynamic PET acquisition (∼3 h post treatment) as described above, 0.6 ml of MRI contrast agent (Gd-DTPA) was injected i.v. at 0.6 ml min^−1^ and MRI scans were performed at ∼3, 24, and 48 h after treatment. The evident difference in accumulation of contrast agent between control and PDT-treated tumours is indicative of an overall decrease in vascular perfusion, as well as a decrease in perfusion over time. These data are also consistent with a vascular-targeting effect following immediate light irradiation after i.v. administration of the photosensitiser (Figure 5).

## Discussion

Our previous studies have shown the ADPM family of photosensitisers to be an exciting new class of agents having significant potential for preclinical development (Gorman *et al*, 2004; Gallagher *et al*, 2005). Unlike many other PDT photosensitisers, ADPM agents are synthetic and can be readily modified about the core periphery to affect drug properties (McDonnell *et al*, 2005). In this study, we have shown our lead agent, ADPM06, to be well tolerated and highly efficacious *in vivo*, when a short drug–light interval protocol was used. Moreover, we have also shown a classical PDT-associated light and photosensitiser dose-dependent response in terms of efficacy, as well as evidence of anti-angiogenic effects *in vitro*. The observed cure rates (>70%) in a xenograft model of breast cancer are encouraging and comparable with the leading vascular-targeting PDT agents currently in clinical development (Trachtenberg *et al*, 2007, 2008). Similar therapeutic responses to treatment were also observed in the LLC/1 Lewis lung carcinoma and 13762 MAT B III breast cancer rodent models.

We have also shown that ADPM06 has an organ biodistribution/clearance pattern consistent with that of an ideal photosensitiser (Chen *et al*, 2006). Using the innate fluorescence properties of ADPM06, its biodistribution pattern in major organs after parenteral delivery has been established. Drug-related fluorescence was undetectable from all organs within 48 h after treatment. There was no apparent toxicity associated with any of the major organs after examination by a veterinary pathologist, indicating no adverse effects of treatment. Importantly, we were unable to detect significant accumulation of drug in the skin of animals 24 h after i.v. delivery of drug, suggesting that patients receiving ADPM06 would be unlikely to suffer the effects of unwanted photosensitiser-induced skin photosensitisation.

Dynamic PET has been previously used to investigate photosensitiser *in vivo* mechanism of action (Lapointe *et al*, 1999; Berard *et al*, 2006a, 2006b). Accordingly, this technology was exploited to investigate the mechanism of action of ADPM06. Dynamic PET profiles, which measure the transient metabolic status of the tumour, provided some initial evidence towards a probable vascular-targeting response after PDT with ADPM06, when a short drug–light interval treatment regimen was used. As rapid destruction of tumour tissue after ADMP06-mediated PDT makes histological assessment of vascular effects difficult, we sought to further examine vascular perfusion effects using MRI. This imaging modality has previously been successfully used to investigate photosensitiser vascular-targeting mechanism of action both in the experimental (Gross *et al*, 2003; Huang *et al*, 2006) and in the clinical context (Haider *et al*, 2007). Our data indicate that vascular perfusion is significantly decreased within 3 h after ADPM06-induced treatment. This effect is escalated over time and is consistent with a vascular-targeting effect when light activation is performed whilst photosensitiser is contained within the vasculature. Direct assessment of the vascular status with MRI also provides crucial information for the interpretation of PET data. Indeed, the PDT vascular shutdown confirmed by MRI essentially discards direct cellular effects as the cause of the metabolic activity drop observed in the PET images. Most recently, blood oxygen level-dependent functional MRI has been applied to further probe PDT mechanism of action in the *in vivo* setting and, most importantly, to determine vascular-targeting effects after treatment (Seshadri *et al*, 2008).

When compared with the historical gold-standard, that is, clinical PDT protocols that allow tumour cell accumulation of photosensitiser before light activation, it has been proposed that the anti-vascular strategy comprises the following benefits: First, blood oxygen levels are known to be the highest at the vascular site in comparison to surrounding tissues, and sensitiser levels in the blood are maximal shortly after injection – an optimal situation for successful PDT. Second, the vascular endothelial cells are directly accessible to the blood-borne sensitiser molecules. Third, it is likely that vascular rupture and haemorrhage formation facilitate rapid discharge of the sensitiser molecules into the tumour site more efficiently than expected by passive diffusion across capillary walls (Chen *et al*, 2006). Tumour cell killing is achieved by oxidative insult during illumination and indirectly by subsequent oxygen and nutrient deprivation due to blood stasis resulting in hypoxia, necrosis, and tumour eradication (Chen *et al*, 2006). These factors are proposed to synergistically contribute to the high success rate of the vascular-targeting treatment protocol.

In summary, we have shown that our lead novel photosensitiser, ADPM06, elicits an impressive therapeutic response in tumour models and is well tolerated *in vivo*. The drug quickly clears both from the skin and internal organs as shown by optical imaging. Application of a combined PET/MRI approach provided evidence for a vascular-targeting response to therapy with this agent. Both imaging modalities may be further used during clinical evaluation as useful biomarkers of therapeutic response. Taken together, the preclinical data set presented here clearly establishes lead BF_2_-azadipyrromethene ADMP06 as an exciting new photosensitiser for potential application in a Phase 1 clinical trial.

## Figures and Tables

**Table 1:** Relative uptake of ^18^F-FDG within treated *vs* control tumours over time

**Time**	**^18^F-FDG relative uptake**
Pre-PDT	1.15
30 min	0.65
24 h	0.33
48 h	0.20

Abbreviation: PDT=photodynamic therapy.
